# Examen Clinique de l'Hydrothérapie

**Published:** 1846-10

**Authors:** 


ART. X.

1. *Examen Clinique de l'Hydrotherapie.* Par H. E. SCHEDEL, Docteur en Médecine.—*Paris,* 1845.
2. *The Dangers of the Water Cure, and its Efficacy examined.* By JAMES WILSON, M.D., and JAMES M. GULLY, M.D.—*London,* 1843.
3. *The Cold Water Cure: its Use and Misuse examined.* By HERBERT MAYO, M.D., F.R.S., formerly Surgeon of Middlesex Hospital.—*London,* 1845.
4. *A Medical Visit to Graeffenberg.* By Sir CHARLES SCUDAMORE, M.D., F.R.S.—*London,* 1843.
5. *Hydropathy.* By EDWARD JOHNSON, M.D.—*London,* 1843.
6. *Graeffenberg; or, a True Report of the Water Cure, with an Account of its Antiquity.* By ROBERT HAY GRAHAM, M.D.—*London,* 1845.
7. *Life at the Water Cure, or a Month at Malvern.* By R. J. LANE.—*London,* 1846.
8. *Confessions of a Water Patient.* By Sir E. B. LYTTON, *Bart.*—1845.

IN consequence of the modern water-cure having been originated by a non-medical and uneducated man, and having been subsequently, for the most part, adopted and professed by lay practitioners, or by medical men of somewhat equivocal reputation,—and yet more, from the system being held out as a *panacea* or cure for all diseases, with an exclusive scorn of medicinal aid,—the medical profession, as a body, have naturally enough, and not inexcusably, treated it with much contempt, not to say aversion, and have shown a pretty general determination not to admit it into the catalogue of therapeutic means. Exercising a natural influence on the public, medical men have succeeded in communicating to a large portion of the intelligent classes the feelings entertained by themselves. Thus hydropathy has become a tabooed subject, being either entirely excluded from medical journals and medical books, or only admitted into them for the purpose of being ridiculed or utterly denounced. Indeed, it is regarded almost as a violation of professional etiquette to mention this subject in the language of toleration, much more to speak of it with approbation. Accordingly, we think it not unlikely that some of our brethren, and those even of the most estimable, may regard our present article as a departure from what is medically proper, and will pronounce us almost worthy to have the severe sentence of "water-doctor" passed against us. We have, however, been too long accustomed to speak our opinions openly and boldly, when we believed them to be just, whether they were in accordance with the current notions or not, to be deterred, on the present occasion, by any apprehended risk of offending mere professional conventionalism. Whatever we conscientiously believe to be true in medical science, especially if, at the same time, calculated to promote the great end and aim of all professors of the healing art—the increase of the means of lessening the sufferings of mankind—that we shall freely and fearlessly promulgate, careless of personal consequences.

Our purpose, in this article, being carefully and calmly to investigate the real merits of the system now so widely established under the name of hydropathy, we hold ourselves absolved from mixing up this investigation with any considerations whatever respecting the merits or demerits, the objects or motives, of those who practise it. We regret to think that there is, and has been from the beginning, not a little quackery and mystification mixed up with really effective practice, in hydropathic proceedings, and that not a few of the conductors of the water-establishments have been, and are, very ill-qualified to indicate, much less to direct and conduct, any therapeutic processes capable of modifying, in an important degree, the vital conditions and functions of the human body. If it shall appear, however, as we believe it will, on further examination, that the external application of cold water is capable of being beneficially applied, in the cure of diseases, in modes of greater efficacy, and to a much greater extent, than has been hitherto practised by medical men, there remains only one course for the members of the profession to pursue, viz. *to adopt the improvements*—if such they are—regardless of their origin, or their past or present relations. When the religious reformer proposed to adapt profane airs to church psalmody, saying that he saw no good reason why the devil should have all the good tunes to himself, he is generally supposed to have acted as wisely as he thought shrewdly and spoke quaintly. In like manner, we see no good reason why the doctors of the orthodox or legitimate school, should refuse to accept good things, even at the hand of the hydropathists. They have done like things before now, as the pharmacopœia, in more pages than one, can testify; and we have not heard that there has been any great reason for regretting that they did so. For our own parts, we avow ourselves of such a catholic spirit, and so lowly-minded withal, as to be ready to grasp any proffered good in the way of HEALING, whosoever may be the offerers, and wheresoever they may have found it. Not merely hydropathy, but even mesmerism, yea, stark-naked and rampant quackery itself, may, in this sense, be a welcome knocker at the gate of physic. It is not the demerits of the donor or the birthplace of the gift, that, in such a case, we are bound to look to—but simply whether it is qualified to aid us in our glorious and divine mission of soothing the pains of our fellow-men. If it is so qualified, the baseness of its source will be lost in the glory of its use; and, if aught of its original impurity still attaches to its application in our hands, the fault will be in us, not in it. A saint may sing the devil's tunes without contamination; a hero may wield the weapon he has wrested from a robber or a murderer; the medicament or the formula of the most arrant quack may be hallowed in the prescription of the true physician.

It is in this spirit we enter upon an investigation of the claims of

Hydropathy, as propounded and practised by Preissnitz and his disciples. And we invite our readers to follow us in a like temper, convinced that they will be benefited by an examination of the subject, whether they adopt our views or not. Some of our views we are sure they must adopt—particularly this: that cold water, applied in the manner of the hydropathists, is a powerful modifier of the condition of the human body, both in health and disease, and, when weighed in the therapeutic balance with other remedies, merits, at least, a fair trial in legitimate practice.

It will be an after consideration in what manner, or under what circumstances, this trial can best be made; and, supposing the result of the trial to be satisfactory, it will be a yet further consideration, and one of great importance, how the remedy shall best be applied in the ordinary practice of medicine. We ourselves believe that distinct bathing establishments will still be found best for giving full effect to the hydropathic system, although we believe, also, that many parts of it may be adopted in ordinary practice at the patients' own homes; and the whole of it certainly be conducted at the water-establishments under the authority and general direction of the ordinary medical attendants. If hydropathy is, as we believe, a therapeutic agent of great power and value, it would be worse than absurd to exclude it from legitimate medicine; but, if it is to be adopted by the profession, it can only be adopted in a strictly professional manner. If distinct establishments are found to be requisite for its complete and successful exhibition, the members of the medical profession can, of course, sanction and patronise those only which are conducted by legally qualified and competent practitioners. And they cannot be expected to show any countenance, even to those which, although under the superintendence of legally qualified persons, are conducted on empirical or absurdly exclusive principles. A hydropathic establishment should be simply a great bathing establishment, or water hospital, and should contain the means for using water in all its medicinal forms, hot as well as cold, in the form of vapour as well as liquid, medicated as well as pure. In such an hospital, although drugs would, doubtless, be but in slight requisition, it would be contrary to all rational proceedings to exclude their use entirely. The very fact of a case being sent to such an hospital, presupposes the previous failure of drugs, or, at least, presumes their unsuitableness in that particular instance; and they would, for the most part, be dispensed with in the *commencement* of the treatment, at least: but no unprejudiced and competent observer can assert that drugs should be entirely banished from the treatment of any case at all times. The same scientific judgment and the same practical skill that prescribed the water-treatment as best calculated to fulfil the indications present at any one time, could alone determine whether, at any other time, medicaments might be proper, either as auxiliaries or substitutes. Nothing but the blindest dogmatism or the wildest empiricism could maintain that, because the water-treatment is found useful, all other means must be useless; or, reversely, that, because drugs are often found beneficial, that therefore all other kinds of treatment, hydropathy included, must be injurious. The absolute exclusionist, be he water-doctor or drug-doctor, is equally unreasonable and equally unjustifiable.

In the composition of the following article we have derived our materials mainly from the published writings of hydropathists, but also, partly from personal observation of the practice of hydropathy itself, and from the reports of patients who had been the subjects of it. We have been careful to select as our authorities the best informed and most impartial of the writers on the subject of the water-cure, and we have used our best endeavours to appropriate what alone seemed trustworthy. It is so extremely difficult for a writer, on any one side of a question that has become the subject of active controversy, to avoid partiality in relating events and drawing inferences, that we make no apology to our authors for having on many occasions refused their evidence and rejected their conclusions. Many things, however, we have admitted on the authority of the writers alone, when they did not seem to be contradicted by other facts, and were in accordance with the general principles of physiology and therapeutics. We have so far admitted the validity of the maxim—*cuilibet in suâ arte credendum;* and, so qualified, we think the propriety of the admission will not be gainsaid. But we have gone further than this. We have accepted at the hands of our hydropathic authors more than one alleged fact and explanation, even although their validity seems to us questionable. And we have done this, because the statements are of a kind justly to challenge attention, and to demand thorough investigation.

On the whole, then, we wish the reader to be prepared to find in the following article, not simply an exposition of the doctrines of hydropathy, as they appear to ourselves well established, but such also as they are laid down by the best authorities of the water-school; one of our objects in writing it being, not merely to endeavour to ascertain what we consider as truth, for the benefit of our readers, but likewise to incite them to make inquiry and examination for themselves, in order that agencies, of such obvious potency on the human frame, may no longer be permitted free scope if evil, or no longer be debarred from ordinary medical practice, if good.

The internal and external use of water, in the treatment of disease, has been frequently discussed by physicians in all ages, from Hippocrates downwards. Their opinions will be found cited in detail by the systematic writers on the subject of baths, and, among others, by Sir John Floyer and Lanzani. To them we refer such as are sufficiently curious to wish for an exact acquaintance with the subject, in its historical relations. For our present purpose, and to render the history of the medical use of water clear to the less minute student, we will group it under a few convenient heads.

1. According to Lanzani,* the true method of using cold water consists almost entirely in its internal administration, in very large doses, in certain stages of certain fevers. His work is most elaborate in every sense; learned, methodical, and comprehensive. It is divided into two books: the first devoted to an explanation of the causes, symptoms, complications, and nature of fever; the second, showing that copious imbibition of cold water is the best means of combating the symptoms, on scientific grounds, and consequently the best remedy for fever. This is obviously an argument somewhat theoretical, but it is supported by a chapter of

* Vero Metodo di servirse dell' Acqua Fredda nelle Febri ed in altri Mali, si interni come esterni. Di Nicolo Lanzani, Medico Napoletano. 2nda edizione. In Napoli, 1723.

cases, and backed by the opinions of a host of learned doctors, the author's predecessors. The actual value of the work is considerably diminished by its scientific character, because many of the doctrines held in its day have now become obsolete, and tend to encumber and obscure, rather than strengthen and enlighten, the practical facts by which they are accompanied. But the same remark applies to the early advocates of other remedies. Lanzani appears to have had no knowledge of the external use of water, nor of its application to the treatment of chronic diseases. He used it in combination with drugs.

Lanzani may be regarded as the representative of a considerable number of writers and practitioners, both in Italy and elsewhere, among whom water has been employed (internally) as the most effectual febrifuge.

2. About the year 1700, Sir John Floyer and Dr. Baynard employed water very freely as an external application, in the ordinary manner of cold bathing, preceding it by a course of physic, and accompanying it generally by copious water-drinking.* Their practice appears to have been chiefly in chronic diseases, such as rheumatism, gout, paralysis, indigestion, general debility, and various nervous affections, in the whole of which a large amount of success is said to have been attained. The baths, at which their cases were treated, were frequently designated by some saint's name. Probably a remnant of superstitious reverence for the saint not only assisted to attract patients to the well, but infused into them a faith in the remedy, which materially promoted their recovery. The practice pursued was simply cold plunging, guarded by certain rules and cautions to prevent accidents.

Sir John Floyer supports his views by the citation of numberless learned authorities, from the Bible to Dr. Mead. He seems to have attached rather an excessive importance to grave precedents, which causes his portion of the conjoint work to savour more of the library than the bedside. At any rate, he mingles together practical facts and the opinions of writers, in such intricate relations, that it is not always easy to discover on which he relies most confidently for the maintenance of his tenets. Dr. Baynard, on the other hand, deals more in cases, of which he presents a very abundant collection. His mode of reasoning is particularly pointed and sagacious. No one can leave the perusal of his work without a strong conviction of his being an honest, shrewd, enterprising, and diligent contributor to medical literature.

These writers mention the occasional practice of persons bathing in their shirts, and wearing them throughout the remainder of the day without drying; they also give an instance or two of cases cured or relieved by the application of a wet towel. The former practice is alluded to as an instance of rashness on the part of patients, and the latter is so rarely mentioned, that in neither can they fairly be said to have anticipated Preissnitz in the systematic employment of the wet sheet or wet compress—although both were actually employed by them. They also speak in very favorable terms of a course of cold preceded by a month's warm bathing, but not

* Psuchrolousia; or, the History of Cold Bathing, both ancient and modern. By Sir John Floyer, of Lichfield, Knt., and Dr. Edward Baynard, Fellow of the College of Physicians, London. 2d edition. London, 1706.

in the modern hydropathic method of the cold following immediately upon the warm, or upon sweating, which is a practice they carefully deprecate. They seem to have had but a slight acquaintance with the use of cold bathing in fever, or acute diseases, though instances of such practice are given.

The following passage from Dr. Baynard, though not strictly a part of our present subject, is a curiosity, and affords a good sample of his peculiar manner. When the period of its publication is considered, it must be regarded, in some of its parts, as a remarkable case of the forestalling of exact experiment by speculative reasoning. Baynard adduces the remarks it contains in support of his hydropathic views; but we need not stay to examine them in that respect. We transcribe portions of the passage:

"*I conceive life to be an actual flame;* as much flame as any culinary flame is, but fed with its peculiar and proper pabulum, made out of the blood and spirits for that purpose; and my reasons are these, viz.: 1. Life is as extinguishable as any flame is, by excluding the air, &c. For hold your handkerchief close to the mouth and nose of any animal that has lungs, and life is put out, the creature is dead in a moment; there is no skin broke, nor bone broke; no wound, nor bruise; there is your whole man, but dead he is. 2d. No flame will burn without aereal niter, or a *quid aerium,* whatever it be; some will have it a mixed gas of niter and sulphur, but whatever it be, 'tis a *causa sine quâ non,* something without which no flame will burn; and that the lungs serve to this use, and are air-strainers, is very clear to me, by that experiment of the candle and two puppy dogs, put into a great oven, and stopped close up with a glass door to see through; and in a little time, when they had sucked in some, and the candle wasted the rest of the niter, the dogs died, and the candle went out with them at the same instant.

"'All ustion, as the *quid inflammabile* wastes, leaves by incineration alkalious and caustical salts, either fixed or volatile, which, from their figure or imbibed fire, become of a pungent corrosive nature, and fix upon the membranes, being nervous and most exquisite of sense and perception, which by irritation cause a light inflammation, which inflammation is called thirst; which salts are melted and washed off by drinking, the grosser by stool with the solid excrements, but those of most solid and subtle particles creep with the chyle into the blood, and have no way out but by the urine. Hence water is the best menstruum to dissolve salts; and that which is most simple and elementary is the best water, as least impregnated; such waters wash off and dissolve their points and angles, by which they prick, sheath, and envelope them in their own pores, and with themselves run off by urine; but if so forced by heat and motion as to disturb them in their passage, the current of urine is checked, and the salts leave their hold of the water, shoot their vortex, and from the channels get into the habit of the body, which, if not dissolved, melted, and thrown off by sweat, they inflame and cause fevers, &c.; nor will they cease their action and inquietude until totally dissolved, or forced back into their common passages, and the salts precipitated and run down by urine. For I look upon the pores and sweat-vents as so many back doors and sally-ports, by which nature drives out the enemy crept into her garrison. This truth is demonstrated in all fevers, where the caustical salts are not washed off, but remain behind on the glands and membranes, forsaken of their dissolving menstruum, the water, &c., *which that ingenious chemist, Mr. George Moult, by chemical analysis made appear in six quarts of febrile urine, which I sent him, and he found but the thirtieth part of those salts usually found in a sound man's urine,* so that of necessity they must remain behind and be left (like so many French dragoons) to quarter on the blood and spirits at discretion. The history of which is printed in the 'Philosophical Transactions' for some years since.

"Now, that which we call an insensible perspiration is nothing else but the smoke made from this vital flame, and the pores are the spiramenta through which it passes, and when these are stopped, this smoke is returned and the flame becomes reverberatory, which is sometimes necessary to force an obstruction, for the body has its registers and vent holes, as well as other furnaces. But to proceed, these salts sometimes crystallize, so that the common menstrua will not touch them, no more than a file will steel or hardened iron, and then it is a true diabetes (and here the physician is at his wit's end, and that no far journey,) then hey ! for lime-water, quince wine, and other restringents, which, if it were possible, would rather make a coalescence, and tie the knot the harder. No; the cure lies in solution by melting down the salts, which must be done by open, raw, and unimpregnated menstrua, such as the Bristol waters are, as most simple, having least contents in them." (pp. 47 et seq.)

3. About the beginning of the present century, Dr. Currie's practice in fever is well known to have consisted principally of cold affusion, or immersion, in the early stages of the disease, and in certain acute affections of the nervous system. His work is so well known that it is unnecessary to enter into any detail as to its contents.* He seems to have known but little of the application of cold water to the treatment of chronic diseases, as represented by Floyer and Baynard, nor to have employed the copious libations described by Lanzani. He cannot be said to have forestalled Preissnitz in any other respect, than in the prompt and energetic use of cold water in the suppression of acute febrile and nervous affections. He brings a large amount of scientific argument and practical experience to bear out his views. He has also placed in a clear light some points of practice on which important errors previously prevailed, such as the safety of cold applications when the body is heated beyond the natural degree, and the relative value and safety of cold or tepid water, of immersion, affusion, and ablution. On these points his work is of great practical value. We may have occasion to revert to some of them hereafter.

4. The prevalent opinions of medical men in this country, on the general subject of the external use of water, previously to the Preissnitzian era, may be considered to be represented in the article Bathing, in the 'Cyclopædia of Practical Medicine,' published within the last twenty years. The article in question places bathing in a very subordinate position among means available for the actual cure of disease. In its cold form it is recommended as a valuable tonic, used with many restrictions, in nervous debility and other analogous states ; and, in its warm form, its use is almost limited to the allaying of irritation in certain disorders, the more formidable symptoms of which are to be encountered by other remedies. Other articles in the same work have done justice to Dr. Currie's views. Beyond this, the medical profession have hitherto done little or nothing with bathing as an instrument of cure. We shall hereafter find reason for believing that a vast superfluity of caution has existed in the employment of this remedy, and that some of the supposed cautions have really increased, instead of diminishing, the danger, as well as destroyed the efficiency of its application.

The author of the article in the Cyclopædia describes cold bathing as partially or absolutely contraindicated in the following conditions : par-

* Medical Reports of the Effects of Water, cold and warm, as a Remedy in Fever and other Diseases. By James Currie, M.D., F.R.S., Fellow of the Royal College of Physicians, Edinburgh. 2 vols. 2d edition. London, 1805.

tially, in infancy and old age; pregnancy; indurations, obstructions, or chronic inflammations of internal parts; acute inflammations of the same; chronic inflammations of mucous membrane: absolutely, during menstruation; in great plethora, or tendency to active hemorrhage, or congestions in important viscera; affections of the heart; loaded state of the bowels; great general debility—though then often advantageous after warm water or vapour bath.

5. The ancient Romans were accustomed to produce perspiration by surrounding the person with heated aqueous vapour, and, while freely perspiring, to plunge into cold water. Interesting remains of baths for this purpose, evidently of Roman architecture, and containing fine specimens of mosaic pavement, may be seen in several parts of England; as, for example, on the margin of the Cranham woods, in the village of Whitcombe, about six miles from Cheltenham; at Bignor in Sussex, &c. It is also well known to have been a frequent practice of the Roman youth to plunge into the Tiber, when heated by exercise in the Campus Martius.

The modern Russians, also, as is well known, excite perspiration in a similar manner, and then roll themselves in snow. A somewhat similar practice has prevailed among the North American Indians. The following description of their process was given by the celebrated Quaker, William Penn, to Dr. Baynard.

"I once saw an instance of it, with divers more in company. For being upon a discovery of the back part of the country, I called upon an Indian of note, whose name was Tenoughan, the captain-general of the clans of Indians of those parts. I found him ill of a fever, his head and limbs much affected with pain, and at the same time his wife preparing a bagnio for him. The bagnio resembled a large oven, into which he crept, by a door on the one side, while she put several red-hot stones in at a small door on the other side thereof, and then fastened the doors as closely from the air as she could. Now, while he was sweating in this bagnio, his wife (for they disdain no service) was, with an axe, cutting her husband a passage into the river (being the winter of '83, the great frost, and the ice very thick), in order to the immersing himself, after he should come out of his bath. In less than half an hour he was in so great a sweat that, when he came out, he was as wet as if he had come out of a river, and the reek or steam of his body so thick, that it was hard to discern anybody's face that stood near him. In this condition, stark naked, he ran into the river, which was about twenty paces, and ducked himself twice or thrice therein, and so returned (passing only through his bagnio to mitigate the immediate stroke of the cold) to his own house, perhaps twenty paces further, and wrapping himself in his woollen mantle, lay down at length near a long (but gentle) fire, in the middle of his wigwam, or house, turning himself several times, till he was dry, and then he rose and fell to getting us our dinner, seeming to be as easy, and well in health, as at any other time." (Baynard, pp. 103-4.)

The extraordinary revivifying effect of the cold plunge bath, after the system has been excited by artificial heat, is testified by various evidence of the most unquestionable kind. Numerous travellers have spoken of this very enthusiastically; among others, Stevens, the American (in his 'Incidents of Travel'), who took a Russian bath, after a most fatiguing journey, and came out of it, he says, quite a new man. We have had similar information from more than one private source. This practice, however, similar as it is to that of Preissnitz, has never, until his time, been extensively, if at all, employed in Europe as a means of curing disease.

6. In the foregoing synopsis are contained the principal forms in which cold bathing and water drinking have been used in the treatment of disease, before these means were so vigorously adopted by Preissnitz. It will be obvious that, from none of the writers mentioned, could he have learned his bold and comprehensive practice. In his method are combined those of Lanzani, Floyer, and Currie, accompanied by novel and powerful processes, to which those writers were entire strangers. The douche, the wet sheet, the sweating blanket, the cold plunging after sweating, the wet compress, the sitting bath (sitz-bath), must be allowed to be, in a great measure, peculiar to the Graeffenberg peasant and his disciples. From the same source have proceeded some important precepts on the subject of diet and regimen. Preissnitz, moreover, is distinguished from all the authorities quoted, by his entire abandonment of drugs.

Vincent Preissnitz was originally a small farmer, residing at Graeffenberg, near the town of Freiwaldeau, in Silesia. He is about fifty years of age. The following is a description of him by Sir Charles Scudamore:

"Of Preissnitz himself I shall say a few words, and describe my impressions on first seeing him. His countenance is full of self-possession; rather agreeable; mild, but firm in expression; with an eye of sense, and a pleasing smile. The smallpox, and the loss of some front teeth from an accident, impair his good looks. His manners are sufficiently well-bred. On closer acquaintance, you discover that he is quick in perception; is reflective; prompt, however, in decision; simple and clear. He inspires his patients with the most entire confidence, and he exacts implicit obedience." (A Medical Visit to Graeffenberg, pp. 2-3.)

Other travellers give a similar description of Preissnitz. They all agree in stating that he is a most arbitrary and tyrannical despot, issuing laws as irrevocable as those of the Medes and Persians, commanding obedience with a haughtiness that might well excite admiration and envy even in an autocrat, and exciting as much fear in his patients as is found in the subjects of the Grand Turk himself. He is also represented as remarkably cool and collected in emergencies, ever ready with his remedy on occasions of danger, and possessed of an imperturbable self-reliance. These traits suffice to prove that he is a man of original and powerful mind, exactly adapted to carry out a novel and startling practice. While his firm and decided manner is calculated to secure the confidence of his patients, his coolness and self-reliance enable him easily to bear the responsibility by which such confidence is attended.

His practice originated in a succession of trifling accidents, by which he was led to employ bathing in a neighbouring spring for the relief of disease. It is not necessary to give them in detail. Success in these first attempts procured him a local renown, and he became the village doctor. From villagers his fame soon spread to patients of a higher rank, and Graeffenberg gradually became the resort of the hipped and the halt from all the surrounding district. By these his praises were sung louder and louder, until all the world began to furnish him patients by the hundred. He now possesses an enormous establishment, capable of containing several hundreds of patients, which is almost constantly crowded with ladies and gentlemen of every degree, and from every nation: while his disciples and followers, as is well known, have spread themselves throughout the world, and maintain, in every country, numerous and flourishing establishments formed on the original model of Graeffenberg.

His treatment, although apparently constructed of such simple elements, is capable of being varied almost *ad infinitum,* according to the peculiarities of the case or the fancy of the prescriber, and of being rendered so powerful, as often to excite in the patients and spectators apprehensions of danger—and sometimes, no doubt, to produce it in reality. It is scarcely too much to say that he has modified the application of water, and some very few other means, in a manner so ingenious as to render them no imperfect *nominal* substitute, at least, for most of the drugs in the pharmacopœia. He has his stimulant, his sedative, his tonic, his reducing agent, his purgative, his astringent, his diuretic, his styptic, his febrifuge, his diaphoretic, his alterative, his counter-irritant. Combined with these are peculiar regulations as to diet, dress, and regimen. The following is his general mode of proceeding.

In his first interview with the patient, after hearing sufficient to give him a rude insight into the locality and general features of the malady, Preissnitz proceeds to investigate its suitableness to his method of cure. He does this by sprinkling the surface of the body with cold water, or witnessing the taking of a cold bath, and then watching the development of reaction. If this appears in a certain amount of activity, he pronounces the case appropriate for his treatment; if not, he advises the abandonment of all hydropathic intentions. This is a mode of ascertaining the power of the constitution quite original, and it cannot be said to be unscientific. The power of resisting the external application of cold is a most essential conservative property of the animal system, and the degree to which it exists must be regarded as, in some respects, a criterion of the amount of *vis medicatrix* possessed by the patient. We see no very decisive reason for pronouncing it a more fallacious guide than the orthodox custom of feeling the pulse. The only objection to it that occurs to us is that it may not be always free from hazard.

This point being satisfactorily determined, the patient is straightway admitted into the mysteries of the cure. In the first place, he finds himself restricted to a peculiar diet. Every stimulant is absolutely prohibited, from brandy and claret, to mustard and pepper; so, also, are most of the luxuries imported from foreign parts, such as tea, coffee, and every kind of spice. The meals consist of three—breakfast at eight, dinner at one, and supper at seven or eight o'clock. For breakfast, cold milk is the beverage, and bread and butter its only substantial companions. At dinner, there is no other restriction than those above named. Supper is a repetition of breakfast, with the occasional addition of preserved fruit or potatoes. Throughout the day, no warm beverage whatever is permitted, and much of the food is brought to table considerably cooled. As some compensation for these manifold deprivations, the patient is allowed to gratify his appetite with every reasonable variety, and a free abundance, of substantial and nutritious food. He finds it a maxim that generous diet will promote his recovery, the treatment being responsible for preventing surfeit. He no longer finds an embargo laid on fruit and vegetables, and is not expected to dine seven days in the week off dry bread and mutton chops. So that, on the whole, there is perhaps about an equal amount of indulgence and restriction, as respects diet, to a patient coming to the Graeffenberg rules from those of some fashionable physician in London.

In the next place, the majority of patients are directed to enter upon a course of water-drinking, the quantity of water varying from five or six to thirty or forty tumblers in the twenty-four hours. A large portion of this is taken before breakfast, the rest at suitable periods after meals, so as not to interfere with digestion, with frequently a glass or two the last thing at night. Exercise is generally advised at the times of water-drinking, except when this accompanies some other process of treatment imcompatible with it.

A third rule insisted on is, that every patient shall take a large amount of exercise during the day. This is, to some degree, indispensable after the cold baths, as a means of procuring the necessary reaction. Walking in the open air is the mode generally selected, when possible. In case of bad weather, or lameness, other plans are contrived; such as gymnastics, sawing or chopping wood, &c. As a general rule, every patient is required to take a long walk before breakfast. It is a *vexata quæstio,* we believe, among hydropathists, as among doctors, whether the patients should rest or walk immediately after a meal; but the water-doctors generally incline to advise very gentle exercise at such times; and, we believe, properly. The well-known experiments on greyhounds, and such other convincing facts, are counterbalanced, to say the least, by the habits of the working man, who proceeds to his labour as soon as he has swallowed his dinner, and rarely suffers from doing so.

After these preliminaries, and the case being pronounced suitable for the treatment, the next morning witnesses the patient's initiation into more active proceedings. At any early hour of the morning, varying according to the time required for the operation about to be undergone, a bath attendant enters with the formidable machinery for the administration of a rubbing with a wet sheet, a packing in the dry blanket, or a packing in the wet sheet. The first of these processes consists of throwing a wet sheet over the whole person, and applying upon it active friction of a few minutes' duration. A glow is thus excited. The patient then dresses, takes his water, and sets forth upon his morning's walk. The second of the above three operations requires the patient to be enveloped in several blankets, with perhaps the superincumbence of a large feather pillow, until free perspiration is excited, which generally requires a period of about three hours. When the perspiration has continued the prescribed time (from fifteen minutes to an hour, or more), the patient is subjected to some kind of cold bath, either by the wet sheet, as just described, by pouring water over the person from pails or watering-pots, or by taking a plunge bath. This being followed by friction and water drinking, the morning's proceedings are concluded by exercise. Packing in the wet sheet is similar to the foregoing, with the addition, next the skin, of a sheet wrung out of cold water. It is generally of shorter duration, as forty-five minutes or an hour, the object being to excite a glow, instead of perspiration. It is followed by cold bathing, as just described. During the packing, in both instances, some glasses of cold water are imbibed through a tube.

At other parts of the day, other portions of the treatment are applied, such as the sitz-bath, the douche, the shower-bath, head-bath, foot-bath, &c.

The sitz-bath is a tub of cold water, in which the patient sits for a period varying from a few minutes to an hour, or even longer, using constant friction to the abdominal region. The other baths mentioned in this paragraph need no description. These, as well as the former processes, are sometimes repeated during the day. In certain cases the day's proceedings commence with some of them, in place of those previously mentioned. A rubbing with the wet sheet is frequently employed before getting into bed at night.

In fever, from whatever source, the patient is enveloped in a succession of wet sheets, renewed as often as they become warm, for a period varying with the intensity of the case—say, from 30 minutes to 5 or 6 hours. In other similar cases, cold immersion or affusion is employed with the same view—viz. to reduce the morbid heat of the system.

The *umschlage*, or compress, is an essential and seldom-omitted part of the treatment. It is a cloth, well wetted with cold water, applied to the surface nearest the supposed seat of the disease, securely covered with a dry cloth, and changed as often as it becomes dry during the day. It is sometimes covered with a layer of oiled silk, which, by impeding evaporation, prevents the inconvenience of frequent change. This compress speedily becomes warm, and remains so until dry. It is termed a heating or stimulating bandage. In cases of superficial inflammation it is more frequently changed, so as to be kept cold, whereby its effect is just the reverse, being then a local antiphlogistic.

In some establishments the sweating has been effected by other means than the simple envelopments of Preissnitz, as by the vapour-bath, or a chamber highly heated by a stove. We have heard of a temperature of 180°, and even that of 198° Fahrenheit, being employed for this purpose. The blankets used by Preissnitz are very bad conductors of caloric, therefore they cause the heat given off by the body to be accumulated around its surface, by the lengthened influence of which the sudorific action is effected. This process differs in no other manner than in degree and rapidity of effect from exposing the same surface to heat of any other origin. The animal heat, when once evolved, becomes a quality of the surrounding atmosphere. Being kept in contact with the body by blankets, it constitutes an artificial elevation of temperature, and nothing more. Therefore, in cases where active sweating is required, we can suppose no disadvantage to result from using other kinds of artificial heat, and can easily imagine advantages in a higher temperature than that attainable from animal heat alone. But we would limit this remark to dry heat. Aqueous vapour, by a well-known law, impedes evaporation, and would therefore restrict the full completion of the sudorific process. For this reason it is used to prevent plants from parting with their moisture in hot-houses. For the same reason it should *not* be used when the intention is to promote the removal of moisture, or to promote perspiration.

A point uniformly insisted on by Preissnitz is, that his patients should abstain from wearing flannel next the skin. When we consider how generally the use of this article of clothing has been advised by physicians and adopted by invalids, especially in this country, we can easily conceive that strong prejudices will exist in the minds of patients against relinquishing it. Yet it appears to be almost universally discarded by hydropathists, and, as far as we have learnt, without any mischievous consequences.

Another maxim of Preissnitz is, that his patients are never to take any kind of drug. It should be remarked that, not being licensed to practise medicine, it would be illegal for him to administer drugs. So that it does not follow, from his disuse of them, that he himself would be opposed to their use *in all cases,* much less that their use is in any way *inconsistent* with his practice. His medical disciples, not being similarly restricted, so far as we can learn, usually employ drugs occasionally, though sparingly.

How are we now to proceed, in order to arrive at a just appreciation of the value of the means thus briefly enumerated? The more usual course would be to enter upon an examination of the practical results, as published by hydropathic writers. But, in the present inquiry, this plan would scarcely answer; for the means employed are so strange, so much at variance with those by which disease is commonly treated, and not a few of the reporters are so little entitled to claim credit for even a capacity to report medical results truly, that the greater part of our readers would disbelieve the alleged facts, rather than admit the principles they would carry with them. It will be more proper, therefore, to omit matters of evidence for the present, and to see if we can find in hydropathic practice any conformity with the principles on which we should estimate the merits of any other new remedy.

If a new vegetable were imported, or a previously unknown chemical substance discovered, and we were called upon to use it as a medicine, we should first inquire whether it possessed any of those qualities which are regarded as constituting medicinal virtues. We might assume that we are sufficiently acquainted with the characters of most diseases, to pronounce what description of influence would have a counteracting effect upon them. It would then remain to inquire, whether the qualities possessed by the article in question were of a kind to lead us to expect any description of such influence from their operation. If they were not, we should be indisposed to try the remedy until well assured, from abundant and unquestionable practical evidence, of its curative powers. If they were, we should be inclined to give it a trial, even if the proofs of its remedial properties were not unexceptionable. For instance, if the article under consideration merely possessed a nauseous taste, a specific colour, or a powerful odour, it would offer little inducement for an experiment of its medical powers, because those qualities are not known to possess any intrinsic influence over any diseased condition. But, if it were a purgative or a sedative, no one could hesitate to recognize it as *a priori* entitled to a trial by physicians; because experience has taught us that, by the means of purging or tranquillising, certain diseases or morbid symptoms may be cured or relieved. And, since it is the case with many of our present remedies, that with the property we wish to employ is combined another we would gladly avoid (purgatives being debilitating, sedatives narcotic, &c.), and with their amount of usefulness is thus associated a certain tendency to mischief,—if the new remedy presented to us appeared to possess the essential quality, and to want the mischievous power of that otherwise used for the same purpose, we should be still more desirous of availing ourselves of it in practice.

If we apply these remarks to Hydropathy, as practised by Preissnitz, the first inquiry ought to be, does it furnish the physician with instruments which he, as a skilful workman, can undertake to employ? Does it contain, among its various machinery, any really therapeutic means, any powers capable of carrying out the indications which we regard as palpable in many diseases? Can it evacuate, can it brace, can it tranquillise? We cannot entertain the idea that the professors of hydropathy have hit upon any grand secret concerning the origin or nature of diseases, or the philosophy of their removal. Such a supposition, were it a necessary article of faith in the hydropathic creed, would render us the most obstinate of sceptics. But, if the practitioners of this new school profess merely to have introduced more efficient, or less dangerous, means of fulfilling the purposes which all physicians have in view in treating disease, we are willing to give them a patient and impartial hearing. Or, if they profess nothing of the kind, and reject such an idea with contempt,—if, nevertheless, their system appear *to us* of the nature we are indicating, we can still entertain it with the hope of discovering something of good in it.

Let us now inquire, then, on physiological and pathological grounds, supported by some personal experience, what appear to be the effects, or among the effects, of a course of water treatment, according to the Preissnitzian system.

1. In the first place, we remark the careful withdrawal of all stimulants from internal parts. In this hydropathy is at once distinguished from ordinary practice. The refinement of civilized life, and the complicated affairs of society, prevent the human frame from being treated entirely as a machine. The body is compelled to undergo an usage not always suitable to its welfare, in consequence of its having to minister to the mind. The exhaustion of the latter, from exertion and excitement, is restored by artificial stimuli applied to the former. These are generally directed to parts ill adapted for their reception. Thus, the stomach, constructed to digest simple food, and to admit fluid at the impulse of thirst, becomes the vehicle of conveying to the nervous system alcohol in its various forms, and other similar fluids. These are unnatural to the stomach itself, though grateful to the nerves. Consequently, the mucous lining of the alimentary canal may suffer in the attainment of an object required only by the nervous system. This is, possibly, the very origin of a proportion of those manifold chronic ailments known under the terms dyspepsia, hypochondriasis, bilious affections, &c., and is unquestionably an aggravating cause in many. To the treatment of these affections the physician brings his purgatives, his carminatives, his anodynes, his stomachics. But it is to the surface of the same unfortunate membrane that they are all applied; and it frequently results, that, when they relieve temporary suffering, they often leave the general health worse than they found it. From this predicament hydropathy professes to be entirely exempt, by abstaining from artificial interference with internal mucous membranes.

2. In the next place, the Hydropathists adopt a system of diet, such as other practitioners seldom venture to prescribe. If a person, suffering from constipation, or any of its long train of attendant ills, applies to an ordinary physician, he is probably told scrupulously to avoid fruit, pastry, and all vegetables, except, perhaps, a favorite one, or, it may be, two. He is also cautioned against the use of veal, pork, even beef, and new bread. We have known such a patient ordered to live for months—we might say, years—constantly on mutton, and bread never less than five days old. This case is neither singular nor infrequent. What is the consequence of this? The patient is compelled to take aperient pills and draughts every day, or every other day; to stimulate the digestive organs (rendered torpid by the use of so monotonous a regimen) by occasional glasses of sherry or porter; and, to compensate the deficient nutrition obtained from so barren a source, by indulgence in strong tea and coffee. Such a patient goes to a hydropathic establishment, and is straightway ushered into a *salle-à-manger*, in which he finds all the variety of food customary at a foreign *table-d'hôte* dinner, and is told to obey the dictates of his appetite. He does so, timidly at first, and apprehensive of direful consequences, but he finds, to his astonishment, than he can take the forbidden luxuries of brocoli, turnips, veal, game, puddings, and fruit, with as much impunity as the never-varied mutton and dry bread, to which he was previously restricted. This is an occurrence so frequently experienced, and so universally attested by hydropathists and their patients, that we cannot refuse to admit it as a point attained by *their system*—therein being comprehended the water *and all its accessories and concomitants.*

3. A third important principle of hydropathic treatment is, that almost all its measures are applied to the surface. It is one of the most formidable difficulties with which the ordinary physician has to contend, that nearly all his remedies reach the point to which they are directed, through one channel. If the brain requires to be placed under the influence of a sedative or a stimulant, if the muscular system demands invigorating by tonics, if the functions of organic life need correction by alteratives, the physician has no means of attaining his object, except by inundating the stomach and bowels with foreign, and frequently to them pernicious, substances. In being thus made the medical doorway to all parts of the system, and so compelled to admit every description of therapeutical applicant, the organ of digestion is contorted to a purpose for which it was never intended. The consequence is, that it has to be consulted before we enter upon the treatment of any case, and it often forbids our availing ourselves of remedies, or plans of action, which are plainly, perhaps urgently, indicated by the condition of other organs, or of the system at large. Thus, to take the three cases above mentioned: how often do we find that one stomach will neither bear ether nor opium; another is injured by steel; and others are intolerant of mercury. The two latter remedies are peculiarly illustrative of these remarks. Iron is employed to raise the tone of the general system, but it occasions constipation by its action on the alimentary canal; therefore, in order to counteract this portion of its effect, it can only be used in conjunction with aloes, or some other purgative, the tendency of which, as respects the system at large, may be exactly the reverse of that of the steel. With mercury, the case is just the opposite. We wish to introduce it into the system, but it is purgative as well as alterative and antiphlogistic, and the former quality often renders very difficult our attaining the benefit of the two latter. The physician, then, is frequently placed in the dilemma, either to injure the stomach in an attempt to relieve other parts, or to leave the latter to their fate, because they can only be rescued at the peril of the former. His only mode of escape from this predicament is, to employ a legion of *adjuvantia, dirigentia,* and *corrigentia,* in the multiplicity and confusion of which it is by no means easy to make out so clear a balance of power as shall enable him clearly to foresee which kind of action, in the *mêlée,* will get the uppermost; and, unless he be well skilled in chemistry, he may unconsciously prescribe a dose so scrupulously guarded as to be neutralized and altogether impotent.

Of course we do not conclude that hydropathy has discovered a remedy for this difficulty; but its own plan of proceeding is not similarly embarrassed, because it deals with outward instead of inward parts. Whether it can produce an efficient substitute for steel, mercury, opium, and other remedies, to which we are alluding, is altogether another question, and one which its professors must bestir themselves to solve, by the careful record and honest publication of their successful and unsuccessful cases.

4. Fourthly, Hydropathy employs a system of most energetic general and local counter-irritation. It has been held by some medical philosophers, that two kinds of morbid action cannot coexist in the same individual. According to this theory, if we can set up an artificial, but harmless, disease by treatment, its development will be attended with the departure of any other disorder that previously existed. Thus is supposed to be explained the operation of mercury in curing various diseases, the disorder arising from its own action being easily disposed of afterwards. We attach no value to this dogma as a dogma, but it serves to embody a large number of well-known facts, and may be as properly appropriated by hydropathists as by other practitioners. By the diligent employment of hydropathic machinery, due regard being had to the constitutional vigour, a condition is often excited, termed by hydropathists *the crisis.* This sometimes consists in the appearance of various cutaneous eruptions; sometimes it is characterized by a series of boils, more or less severe; in other cases its leading feature is disturbance of the function of some internal organ, creating diarrhea, abnormal urinary discharges, vomiting, &c. In general this effect is trifling, and seldom proceeds to such a degree as to excite alarm, or to give cause for special interference; so that the measures which have led to its appearance are in most cases continued, and in some even increased, until it has run through its course and subsided. This is not always the case: sometimes it proceeds to a more serious length, and requires careful management to prevent mischief; the boils, in particular, are frequently very troublesome;—even death has, in a certain proportion of instances, ensued, either as an immediate or remote consequence of the so-called crisis.

Whatever the crisis may be—or whether what is so called *be* a crisis in reality—there is no disputing that it results from the operation of a powerful system of counter-irritation—or of irritation, at least. It is to this that we now wish to direct attention, because we suspect that in it is contained the true explanation of the good effects of the water-cure in many chronic cases.

5. A fifth physiological feature of the water-cure is the number of *coolings* to which the body is subjected during the day. The generation of caloric in the animal system has been traced to its real source. It results from the *burning up* of waste matter, which, by accumulation, would become injurious. The oxygen of the atmosphere, admitted into the lungs by inspiration, traverses the various blood-vessels of the body, and, in the minute capillaries, unites with carbonized substances. The union produces the carbonic acid emitted from the lungs in expiration, and is attended with the development of what is called animal heat. It is obvious that lowering the temperature of the body, within certain limits, by awakening an uncomfortable sensation in the nerves, would induce increased activity in this calorific process, in order to maintain or restore the average degree of warmth. This increased activity could only be supported by an additional consumption of carbonized matter. If the carbonized matter were already there, and if its existence constituted the disease, or an important part of it, as is probably sometimes the case, a perfect cure would result from its removal. But, supposing there is no such matter present, what then would be the consequence of stimulating this decarbonizing operation? The consequence would certainly be, that the constituents of the tissues themselves would be consumed, in order to supply the pabulum required by the oxygen. This would as certainly excite an effort at restoration, by which the digestive organs would aim to renew to the tissues the amount abstracted by the oxygen. In other words, the appetite would be increased.

Hence it is that more food is required in cold climates than in warm—in winter than in summer. The greater consumption necessary to maintain equal temperature in cold weather, can only be met by increased supply. What, in a vague and general manner, arises from the ordinary progress of the seasons, may be rendered methodical and profitable, by the careful interference of art.

It has been urged that the effect here considered would equally result from exposure to cold air, as to cold water. In the words of Mr. Herbert Mayo, "This is not only entertaining, but satisfactory as far as it goes; and admits very well of being popularly and loosely brought forward in favour of cold bathing: but unluckily it is as much or more in favour of our living in Nova Zembla, as of our resorting to Graeffenberg." (p. 5.) The same intelligent writer proceeds to notice other modes of exposure to cold, which are found to produce evil instead of good, which are, indeed, familiar as the frequent causes of serious disease, and against which we are of old cautioned:

"Nudus ara, sere nudus,—habebis frigora, febrim."

It is singular enough that this very argument, now employed to discountenance the use of cold bathing, is the very strongest theoretical argument in its favour, as was long ago pointed out by that sarcastic writer, Dr. Baynard, in the following anecdote:

"Here a demi-brained doctor, of more note than nous, asked, in the amazed agony of his half-understanding, how 'twas possible that an external application should affect the bowels and cure the pain within. Why, doctor, quoth an old woman standing by, by the same reason that, being wet-shod, or catching cold from without, should give you the gripes and pain within." (p. 119.)

If a rude exposure of the surface to cold and wet is capable of producing internal disease, there is no doubt that a close relation exists between those agents and the morbid conditions of internal parts. Therefore, if they could, by skilful management, be so applied as to excite an opposite effect from that to which their bad consequences are due, they would then become equally powerful means of removing disease. This is the very thing that Preissnitz and his disciples profess to have done—and to do.

Let us, then, consider a little further the consequence of repeated applications of cold, supposing, for the sake of argument, it is used with due reference to the constitutional powers, so as to create an increased activity of the vital functions. It appears to us that this is exactly the thing needed in the treatment of a great many cases of chronic ailments. It is easy enough to construct methodical catalogues of organic lesions and their symptoms, and to assign, on paper, a "local habitation and a name" for every malady that is to require our treatment. But the truth is that, practically speaking, there are a vast number of cases in which the symptoms may be said to constitute the only disease that can be detected, and in which they point rather to a general torpidity or derangement of all, or almost all, the vital functions, than to special change or disturbance in any particular organ. Many cases known as indigestion, gout, rheumatism, liver complaints, or nervous affections, come under this description. In a large portion of such cases, and their like, we could conceive the practice of Preissnitz to be peculiarly beneficial, if it consisted in nothing more than the frequent application, and skilful adaptation, of cold water. It was mainly by this means that the cures described by Floyer and Baynard were effected, simple cold bathing having been almost their only instrument.

6. Another physiological feature of hydropathic treatment consists in its creating a large amount of stimulation in the system. This stimulation is of a peculiar kind, and very different from that produced by alcoholic fluids or pharmaceutical stimulants. The difference is in its not awakening abnormal activity, to be succeeded by abnormal depression, in the nerves and organs of circulation, as is done by the stimulants just mentioned. The fall of a heavy douche, the sudden plunging into a cold bath with speedy exit, active friction in a shallow bath, are means of stimulating the system in the manner here intended. The effect, we are told, is manifested in the altered look of the patient after taking the bath, in his freshened cheek, his brightened eye, his elastic step, his cheerful tone. But it is *not* manifested in a quickened pulse, or a heated imagination, nor followed by exhausted energy or lowered spirits. This is the description given by hydropathists (whose practice we are not teaching but describing) —and which we have ourselves heard given by patients. It is also said that drinking, in rapid succession, several glasses of perfectly cold water has a decidedly stimulating influence on the system. If these descriptions be correct of hydropathic stimulants, that they are powerful as well as innocuous, exciting and not exhausting, they constitute a valuable instrument in the treatment of disease, and deserve the more careful attention of physicians.

We happen to have been acquainted with the case of a lady who was at a hydropathic institution for the treatment of very aggravated chronic rheumatism. Her general powers were much shaken, and she had been unable to walk at all for a period of about four years, before undergoing this system of treatment. After several weeks of sweating and cold plunging, locomotion began gradually to return. The first indication of this was, that she could walk a few steps *immediately after leaving the cold bath.* For a considerable time this continued to be the only occasion of her being able to walk during the day, though she afterwards made considerably further progress. We mention this case because we can guarantee its truth, and it always appeared to us a striking and instructive instance of the stimulating property of a cold bath.

7. A still more important and less questionable quality of the water cure is its power of lowering the system to any extent, without any of the debilitating means otherwise used for that purpose. In a general inflammatory or febrile condition of the body, a lengthened immersion in cold water, or envelopment in a succession of cold wet sheets, would reduce the temperature and force of circulation to the most extreme degree. These means are, to the functions of life, what an extinguisher is to a flame. Their reducing power can be gradually applied up to the point of actual extinction. Anywhere short of that, withdraw the means, and the flame, whether of oil or of life, gradually resumes its previous brilliancy.

In the treatment of febrile diseases an important indication is to reduce the morbidly increased activity of some of the organic functions, most distinctly manifested in the circulation and the temperature. For this purpose the great instrument heretofore most in use is bloodletting, as being our only certain and expeditious method of reducing the frequency, force, or fulness of the pulse. So that, in order to suppress febrile action, we hazard occasioning a more or less lingering debility. The *post hoc,* whether *propter hoc* or not, is too frequently a protracted convalescence, during which the patient is in constant danger of relapse. The mortality that occurs during convalescence after fever, from recurrence of the original disease, from some of its numerous sequelæ, or from the accidental inroad of some other disorder, is so considerable as to render this a period of great anxiety to the patient and the physician. It is a question deserving of cautious and dispassionate investigation, whether any portion of the liability to these mishaps is attributable to the bleeding, purging, salivating, and low diet, employed in removing the fever.

In some of the cases of fever described by Currie we cannot fail to be struck by the rapidity and completeness of the cures effected by cold affusion or immersion, when used sufficiently early. The disease appears to have been suddenly checked and destroyed. In the course of a few hours, or a day or two, a patient threatened with, or labouring under, a dangerous fever, was restored to perfect health. No period of debility ensued, no organs were found to have been seriously or permanently injured. The result of his well-known treatment, by cold bathing, of the fever which appeared in the 30th Regiment is thus described:

"These means were successful in arresting the epidemic; after the 13th of June no person was attacked by it. It extended to fifty-eight persons in all, of which thirty-two went through the regular course of the fever, *and in twenty-six the disease seemed to be cut short by the cold affusion.* Of the thirty-two already mentioned two died. Both of these were men whose constitutions were weakened by the climate of the West Indies; *both of them had been bled in the early stages of the fever;* and one of them being in the twelfth, the other in the fourteenth day of the disease, when I first visited them, neither of them was subjected to the cold affusion." (Vol. i, p. 13.)

Again:

"In cases in which the affusion was not employed till the third day of the fever, I have seen several instances of the same complete solution of the disease. I have even seen this take place when the remedy had been deferred till the fourth day; but this is not common." (Ibid. p. 23.)

In contemplating these facts, we are driven seriously to ask, not only is not the debility consecutive to fever partly occasioned by the remedies employed in its treatment, but are not its attendant local and organic lesions in great measure produced by the febrile paroxysm itself? And could they not be avoided, by boldly applying a remedy by which this febrile condition would be more speedily subdued? The real nature of fever is, unfortunately, beyond the reach of our present knowledge. We only recognize the disease in its causes, its symptoms, its complications. In them we perceive much to lead us to answer the above questions in the affirmative. It is peculiarly a general disease. Its local characters usually appear subsequently to its general development, and wear much more the aspect of consequences than of causes. Almost any of the local complications of synochus, or typhus, may appear in exanthematous fevers, where they cannot be causes.

It appears to us to be a most important subject of inquiry whether a very serious fallacy does not pervade the medical profession at present as to the best manner of employing cold water in fever. Dr. Currie says:

"When the affusion of water, cold or tepid, is not employed in fever, benefit may be derived, as has already been mentioned, though in an inferior degree, by sponging or wetting the body with cold or warm vinegar or water. This application is, however, to be regulated, like the others, by the actual state of the patient's heat, and of his sensations. *According to my experience, it is not only less effectual, but in many cases less safe; for the system will often bear a sudden, a general, and a stimulating application of cold, when it shrinks from its slow and successive application.*" (Vol. i, p. 73.)

"It is evident De Haen was not regulated, in his use of external ablution with cold water, by rules similar to those which I have ventured to lay down from several years' experience. Instead of pouring water over the naked body, *he applied sponges soaked in cold water to every part of the surface in succession for some time together, in my judgment the least efficacious, as well as the most hazardous, manner of using the remedy.*" (Ibid. p. 84, note.)

This is a remark which we suspect to be of very great importance, and to contain the real secret of much of the difference, as to the treatment of fever, between hydropathists and the regular faculty. Modern physicians have professed to regard Dr. Currie as a very high authority on this point, and his work is constantly quoted as the most enlightened guide for the use of water in fever; but the above opinion and precept have been, of late years, entirely disregarded, and the converse has been made the rule of practice. In the article on Bathing, in the Cyclopædia, formerly referred to, the author says:

"The only cases in which refrigeration is required as a remedy are those in which the animal temperature is elevated above the natural standard; and this happens only *in febrile diseases.* To ensure refrigeration, the water should be applied *at first only a little below the temperature of the skin,* its heat being insensibly and gradually reduced, *but never below that of tepid, or, at most, cool.* The *gentlest mode of applying it is the best, as with a soft sponge;* and the process should be persevered in, without interruption, until the desired effect is produced." (Art. Bathing, Cyclopædia of Practical Medicine.)

We believe this mode of applying water in the treatment of febrile diseases to be that which has for many years generally prevailed, not from ignorance of the precepts and practice of Dr. Currie, but from a general belief that fever once formed could not be extinguished by the cold affusion as recommended by him. The hydropathists have renewed his system in its full boldness. It is, therefore, a question of the first interest, on which side does reason preponderate?

On carefully examining the cases of fever reported at length by Currie and Lanzani, it will be seen that their cures were effected by what may be termed a process of reaction. The immediate consequence, in most cases, of the copious libation "*ultra satietatem*" of the one, and the affusion, or immersion, of the other, were perspiration and sleep. These constituted the reaction. When exacerbation of the fever ensued, and required a repetition of the remedy, it occurred several hours after the cold application, when the period of reaction had long passed over; and evidently proceeded, not from the consequences of the cold treatment, but from the non-removal of the diseased action. The cold appears to have acted in a most decidedly *medical* manner, with a palpable and immediate succession of consequences altogether different from what the gradual coolness of the sponge and tepid water can be expected to produce. If these cases are correctly stated, as they appear to be, it is preposterous to confound the febrile paroxysm with reaction from a cold bath, or to expect any portion of the beneficial effect of cold immersion in fever, from tepid or cool sponging. The two kinds of treatment are in no measure similar.

But it may be supposed there is a danger in the sudden and active employment of the cold bath in fever. We suspect that this is entirely imaginary. Dr. Currie was certainly very bold in its administration, and had extensive experience of its effects. In the second edition of his work he says:

"I have thus related all the instances which have occurred to me since the last edition of this volume (a period of five years of extensive and attentive observation), in which the affusion of water on the surface of the body, cold or tepid, proved either less beneficial in its effects in fever than I had formerly represented it, or entirely unsuccessful. I would add, if any such had occurred, the instances in which this remedy had appeared to be injurious. But experience has suggested to me no instance of the kind, and extensive as my employment of the affusion has been, *I have never heard that it has suggested,* even to the fears or prejudices of others, *a single occasion of imputing injury to the remedy.*" (Vol. ii, p. 25.)

This statement, which does not appear to have been assailed, goes far towards proving the innocence, as his numerous cases do the curative powers, of reaction in the treatment of fever. We certainly cannot quarrel with hydropathists for seeking to revive, in its real character, a method supported by so high an authority.

8. It is scarcely necessary to remark that a judicious system of cold bathing is a valuable tonic. This has been always known; but it has not been so widely recognized in practice as in doctrine. It has been thought necessary that cases for cold bathing should be carefully selected; that they should consist only of such patients as have unimpaired constitutions; that certain diseases were absolute contraindications against the use of this remedy; that it is a treatment requiring unquestionable vigour in the patient, and skill in the physician to employ it without injury. It is scarcely too much to say it has been regarded as a treatment rather for the strong than the weak, and as tending rather to reduce than augment the powers of the system—and yet it is called a tonic. This is an illogical paradox not quite solitary in medical literature. The cold bath seems to be professionally employed to strengthen the body, as temptation is to strengthen virtue, by furnishing an enemy to struggle against. Thus it is considered more as a test than as a source of strength.

The hydropathists have discarded this excessive precaution, and boldly used their remedy as a tonic, wherever a tonic is required. They have administered it to the young and the old, the weak, the bilious, the gouty, the scrofulous, the dyspeptic, and the paralytic. Neither mucous membranes nor mesenteric glands, infantile weakness, nor senile decrepitude have stood in their way. To almost all cases, all ages, and all constitutions, their method has been applied. Unless it can be shown that this all but universal administration of the system has produced serious evils, we are actually driven to admit that it is in the same proportion safe. And we are bound to admit—though we have known some instances where the practice has been seriously injurious, and have heard of others of a similar kind—that the proportion of bad consequences has not appeared to us greater than in the ordinary modes of treating similar diseases. The practice of the hydropathists is so open, and their disciples so numerous, that the innocence of their proceedings may be said to be established by the absence of evidence to the contrary. We cannot enter any circle of society without encountering some follower of this method, ready to narrate a series of *psuchrolousian* miracles, prepared to defend, and zealous to applaud the Preissnitzian practice; but few or none come forward with satisfactory evidence of anything like general mischief having resulted from its general practice. Judgment must, therefore, be entered by default against its opponents, and hydropathy is entitled to the verdict of harmlessness, since cause has never been shown to the contrary.

But not only have hydropathists despised the discrimination usually employed in the selection of cases for cold bathing, they have manifested an equal apparent boldness in the manner of using it. In place of the spongings and the dribblings to which ordinary practitioners commonly deem it prudent to limit the use of this remedy, they employ active plunging and powerful douches. Perhaps it is to this that they owe some portion of the impunity with which they appear to have applied it so generally. They assert that the more violent practice is really the more safe, and that the danger to be apprehended is in proportion to the supposed mildness of the process, sponging being less safe than total immersion, and a shower-bath more dangerous than a douche. And assuredly theory, in this respect, goes with them to some extent. In the plunge there is a sudden shock, which awakens nervous energy, and leads to speedy and effectual reaction; whereas, in sponging, the whole surface is exposed to a gradual and powerful cooling, without the protection of stimulus. In the former, the whole frame is at once covered with water, and shielded from the reducing evaporation which would attend the latter. Moreover, the plunge can be more speedily gone through, and followed up more immediately by exercise. The same distinction may be made between the hydropathic douche and the orthodox shower-bath. The force of the latter falls almost exclusively on the head and shoulders, as it merely trickles down the rest of the frame. How different is this from the powerful impulse of the douche upon all the muscular parts!

9. Another conspicuous item in the catalogue of hydropathic machinery is the *sweating process*. On this subject hydropathists are, in some measure, divided. It is said that Preissnitz has considerably modified his views respecting its efficacy and its safety. In the earlier period of his practice he seems to have employed it in nearly all cases. More recently he is said to have discarded it, as a general remedy, in favour of packing in the wet sheet, though still largely applying it in cases to which his matured experience has taught him to regard it as especially beneficial. We wish here to direct attention to it merely in a physiological and pathological point of view, and need not, therefore, enter into the question as to the relative value of the past and present practice of Preissnitz.

The skin is a part through which nature has arranged that a large amount of matter should be removed from the body during health, and a still larger amount, of different character, in the process of recovery from many diseases. It is well known that a deficient cutaneous excretion is incompatible with perfect health. Perhaps there is scarcely any disease in which the function of the skin is not, to some extent, deranged. To what extent, physicians have not bestowed sufficient pains to learn; nor have they been accustomed to give much attention to this part, in the practical investigation of diseases. Still less has it acquired an important position in the list of parts to which medical treatment is directed. Therefore, we possess little information, in medical writers, as to the amount or frequency of cutaneous disturbance in general disease, as to the effect of therapeutic means in correcting such disturbance, or as to the value of the correction in the cure of disease.

According to Preissnitzian writers, in almost all cases of indigestion, gout, rheumatism, nervous affections, indeed of chronic disorders in general, the action of the skin is either deficient or depraved, the part itself being found dry, hard, rough, thick, pale, relaxed, or in some other manner unnatural. They farther tell us that a course of perspiration, or of the wet sheet, followed by cold bathing, corrects these signs of disorder, and reduces the part to its normal condition; and that the beneficial influence of the remedy is speedily manifested in the improvement of the case in other respects. But it might be expected that such a course would, at least, reduce the general strength, and require more vigour of constitution than many such patients possess. And yet, if we may believe the hydropathists, or even their patients, a course of active hydropathic sweating is found to strengthen, instead of weakening, the system. There is a gain, instead of a loss, of weight under its operation. Whether this be attributable to the subsequent cold bathing, to the water drinking, or to the peculiar regimen, may be a matter of question; but the fact would seem to be too notorious to be contradicted. We are told that it is no infrequent occurrence at hydropathic establishments for the liquid perspiration to be streaming on the floor, having penetrated through the material on which the patient is reclining, as well as the blanket in which he is wrapped! The blanket also, when removed from the person, is dripping with liquid in all directions, as if itself just removed from the bath! On these occasions several pounds of matter must be removed from the body. The patient, dripping and steaming, next hastens into the plunge bath, stays there his appointed time, undergoes the prescribed friction, drinks his water, and finds himself actually invigorated by the strange process he has undergone!

Nay, more: it is placed beyond doubt, by experience, that this proceeding may be repeated daily, or even twice a day, for *many months,* without producing any deleterious effect upon the general health! Many cases have occurred in which it has been ascertained that it has been attended with an increase of weight, and that of no slight amount. We know the particulars of one case, in which a gouty gentleman gained seven pounds in a fortnight of such treatment; and of another, in which there was a gain of eight pounds in ten days. We are also acquainted with the case of a lady who was unable to walk at any other period of the day, except immediately after the sweating process—a sure proof that it did not occasion debility.

The safety of the immediate succession of cold bathing upon copious sweating has been called in question; but the practice of so many hydropathists as there are around us amply establishes this point. On scientific grounds the question was completely set at rest by Dr. Currie.

An effective and innocuous means of increasing the excretion from the skin being thus found, which appears to combine with its own peculiar action the indirect effect of a tonic, have we not reason to regard it as a promising instrument of cure, in many disordered states of the system? We fully believe that we have. We know the utility of augmenting the secretion of the mucous membranes, the liver, the kidneys; we recognize this in our constant practice. It is by this means that we combat a large proportion of chronic as well as acute maladies. Why should the skin alone be neglected? Physiology teaches us that it is the vehicle for conveying out of the system a large amount of matter, as well solid as liquid; and practical experience exhibits it as the channel through which the *materies morbi* in many instances, and the burthen of plethora generally, find their exit. These facts indicate it as a legitimate locality for the same artificial measures which are found serviceable on other secreting organs.

It may be objected to what we are now urging, that profuse perspiration itself characterizes many diseases, of which it is one of the most formidable symptoms. How can sweating cure acute rheumatism, it may be asked, of which it is almost a constant feature? But the same remark applies to other medical phenomena. Excessive purging and increased action of the kidneys are dangerous, frequently mortal, symptoms. But does that prevent our employing them as remedies? Do we not, in spite of our frequent experience of their injurious effects, apply them almost constantly to the cure of disease? Are there ten cases out of ten thousand in which some kind of purgatives are not administered? Nay, is not dysentery itself treated by purgative calomel? Let us extend the same tolerance to sweating. It is contrary to all the instruction of experience to confound the consequences of a phenomenon violently excited by morbid causes, with those it induces when seasonably created, and carefully managed, by skilful treatment.

In many of these cases, the benefit does not appear to result so much from stimulating the function of any particular organ, as from removing a certain portion of matter from the system at large. There is no reason to suppose that exciting the liver, the colon, the duodenum, or the kidneys, for instance, has any special influence over a morbid condition of the brain. We find that drugs which act upon any of those organs, frequently relieve such conditions, and they may often be selected indiscriminately, the one answering much the same purpose as the others. A common antibilious pill, retailed for a penny by a druggist, or a patented nostrum of Cockle or Morrison, will generally do as well as the most elaborate prescription. The particular adaptation seems to depend more on constitutional idiosyncrasy than on any fixed relation of the part diseased with the part treated. The whole of those remedies appear to act in such cases, either by a general principle of counter-irritation, or by removing a quantum of fluid, or of excretory matter, from the circulation, either of which objects might be attained as speedily, as certainly, as extensively, and as safely, by the skin as by any other part.

10. But the power of the water-cure over excretions is not limited to the skin. It professes to be both a purgative and a diuretic. That it is diuretic, in a certain sense, needs no proof. It is no new discovery that, in proportion to the quantity of fluid imbibed by the mouth will be the quantity emitted by the kidneys. This, though verbally, is not medically a diuretic action. It may consist simply in the mechanical discharge of the fluid imbibed, with no augmentation of the proper functions of the kidneys, as respects the previous condition of the blood-vessels. But it is not perhaps unphilosophical to give hydropathists the benefit of supposing that water-drinking may do indirectly what it does not appear to do directly: by its diluting power, may it not destroy the influence of any mischievous constituent of the blood, the excess of dilution being immediately repaired by the removal of the water through the kidneys, in company with the deleterious matter dissolved in it? This view might be admitted, if it could be shown, practically, that drinking water has the same effect on disease as taking diuretics.

The purgative action of hydropathy is less equivocal. It frequently happens, in cases of constipation, that, after a few days' or weeks' use of its appliances, the patient is attacked with diarrhea. This is sometimes troublesome, but we believe seldom dangerous. On its subsiding, the bowels are said to have generally acquired a regular and healthy action, which is thenceforward maintained by persevering in the drinking, bathing, &c. In other cases, a regular action of the bowels comes on in a gradual manner, without the occurrence of diarrhea, the treatment appearing to influence the bowels through its action on the system at large. In others, and every one has seen examples of this, the mere drinking of a few glasses of water before breakfast is represented as a purgative that may be relied on. In these the daily dose is regulated according to circumstances, being increased when signs of torpidity are observed. We are ourselves acquainted with some persons who regulate this function as accurately by water-drinking as they formerly did by medicinal aperients. There are cases, again, in which the sitz-bath, or other external applications of cold water, produce a purgative effect.

It may be asked, is not this effect too uniform for the purpose of the practical physician? Does it not often result from the mere percolation of water through the mucous lining of the intestinal canal? Is it not, therefore, a mere pouring out of what has been swallowed? Is it not clearly inadequate to excite the particular action of the liver, the pancreas, the lower or upper portion of the intestinal tube? Is it not necessary that we should be able to act on these parts separately, for the effectual cure of disease? These questions, important as they appear, may with equal justice be asked as to the practical proceedings of our profession in general. It is true that, in theory, many nice distinctions are laid down respecting the peculiar operation, as to locality or otherwise, of different cathartics. But are these distinctions generally observed in practice? Did not Abernethy's page 72 contain the curative maxim for all cases? and were not his prescriptions almost always identical? Has not every respectable family doctor his "my pills," carefully prepared of the same ingredients for every difficulty in the bowels? Is not the black draught as universal a purgative as Preissnitz would make cold water? Are not all our moneyed dyspeptics and hypochondriac nabobs sent in a body to mineral springs, because they are purgative, without any preliminary investigation as to their action on the duodenum or the colon, the liver or the pancreas, or as to the expediency of such action in the individual case in question?

11. We observe, also, in the history of hydropathic practice, the development of a peculiar sedative or tranquillising influence. It is well illustrated in the following passage from Mr. Mayo's preface:

"Through repeated attacks of a sort of rheumatism my constitution appeared completely broken down. Already crippled in my limbs, *preserving what power of exertion I still retained only through the use of opium*, and my indisposition still increasing, I looked forward to being before long worn out with suffering—as to death, as a release. I could not bear the fatigue of a land journey, or I should have gone at once to Graeffenberg; but Coblentz and Boppard might be reached from London by water—so I went to Marienburg in June, 1842. On arriving there I was placed on a routine system of sweating and bathing. The immediate effect on my health was strikingly beneficial, *and in a week I was able to relinquish the use of opium*. The rheumatism did not, however, give way proportionably to my general improvement. The pains of the joints were, indeed, heightened." (p. 1.)

This was a painfully severe case, one in which every conceivable remedy had been previously tried, not excepting repeated change of air, the Bath-waters, &c.; yet nothing had succeeded in relieving the system from the necessity of constantly using opium. A "routine system of sweating and bathing" was applied, and in a week the patient was able to relinquish his doses of opium, notwithstanding that the rheumatism did not give way; indeed, the pains in the joints increased. How is this to be explained? only by supposing that, independent of any curative influence over the actual disease, the water-cure exercised some sort of sedative action on the system at large. Similar instances are said to be familiar at hydropathic establishments. If these accounts may be depended on, hydropathy would appear to contain, in its armamentarium even an anodyne, and one of great power. Every practitioner knows the difficulty presented in the treatment of chronic cases, by morbid irritability, and painful nervous sensations, which are not only intolerable to the patient himself, but most prejudicial to his recovery; and which can only be relieved, from time to time, by repeated and gradually augmented doses of a drug, whose own effects are almost as pernicious as the symptoms it is used to palliate. This is one instance of a predicament in which the physician is not infrequently placed, when he has most gravely to consider whether there is most mischief in the disease to be combated, or in the only remedy by which it can be encountered. If "a routine system of sweating and bathing" affords a means of extrication from the present instance of this difficulty, this is a strong reason why it should not continue to be obstinately excluded from the well-fenced pale of the medical profession.

12. In addition to the effects already considered, and which have occupied as much as can be spared of our space, the water-cure pretends to the possession of other important powers. Thus, it is said to be a *stomachic,* since it almost invariably increases the appetite. It is a local *calefacient,* in the application of the wet cloth covered by the dry one. It is a *derivative,* cold friction at one part, by exciting increased action there, producing corresponding diminution elsewhere. It is a local as well as general *counter-irritant,* the compress frequently acting, if not like a blister, at least, like a mustard poultice. It is essentially *alterative* in the continued removal of old matter by sweating, and its renewal as shown in the maintenance of the same weight.

13. Lastly, our subject brings us to make a few remarks on *medical habits* in reference to chronic cases. In such cases, we have only commenced the treatment, when we have removed the immediate symptoms; the real difficulty consists in preventing their recurrence. Accordingly, the patient quits his physician with ample instructions for his future guidance, and with most impressive warnings as to perseverance in their observance. What are these instructions, and to what habits do they lead? Let us take a case of "biliousness" or chronic dyspepsy, and briefly trace the history of its "legitimate" treatment, according to the heroic school of London.

In addition to constipation, the patient, we shall suppose, is affected with acidity, deficient or depraved appetite, foul tongue, oppression after meals, susceptibility to cold, debility, headache, despondency, irritability of temper, inconstancy of purpose, hopelessness of relief, with divers local grievances. A few brisk cathartic doses, combined with mutton diet, and a gentle stimulant, empty the bowels, and carry off most of the attendant ills. By continuing this plan for a short time the patient is, what is medically termed, cured; but, for future protection, he is furnished with a prescription—say—of aloes, colocynth, and calomel, or some such compound, to take *pro re natâ;* another of senna and salts, to take less frequently, as more urgent symptoms require; a third of calumba, gentian, or cinchona, to take at noon with a glass of sherry. He is told to live on boiled mutton, rice, and dry bread, avoiding fruit and vegetables.

What future, as respects health, has such a person before him? As long as he lives he will be a martyr to the disease, probably in an increasing degree; he must abandon all hope of the action of the bowels ever resuming its normal state; his general strength will gradually diminish; his nervous system will become more and more irritable; his whole comfort and enjoyment will be sacrificed in order to empty the alimentary canal; he will become one of those most pitiable of all sufferers, a person "living by rule;" his health will be supported, as one of our witty doctors remarks, like a shuttlecock between two battledoors, by the alternate impulse of senna and sherry, of calomel and coffee, of jalap and gentian. As long as these instruments are so directed, that their respective influences succeed each other in compensating proportion, all seems, for the time, smooth; but let either overdo or underdo the mark, and everything breaks down. The game must then be commenced anew, to be continued as long as feather and cork resist the tendency which it has to knock them to pieces.

This is scarcely a caricatured picture of the discipline to which dyspeptic patients are often forced to submit. Everybody's experience must furnish abundant proof that the illustration is too close to nature. It is in the later stages of these affections, when the patients have long been under the influence of therapeutic means, that Preissnitz pronounces them "drug-diseases." If, by this term, he means that drugs constitute the whole disease, then he is no doubt wrong; yet, in one point of view, he is right. The original complaint for which the drugs were administered might, very probably, have been one requiring some artificial remedy, and which would have induced more serious consequences, had not some such remedy been employed. But it is quite possible that a persevering use of such remedies may create a train of symptoms, in addition to those which existed before, and induce such a host of wants as may constitute a prominent feature of the case, by the time it is submitted, to the curative process of such a practitioner as Preissnitz; therefore his term, drug-disease, may not be altogether inapplicable.

But what is often the result of placing the cases, now under consideration, in a hydropathic establishment? Precisely such as might be expected from the abandonment of a pernicious custom, and the adoption, at the same time, of a more natural mode of life with healthier and hardier habits; and with the immense additional mental stimulus of cheerfulness, of faith and hope in the new system, and of unbounded confidence in the new doctor. It is accordingly the general report that, in a large proportion of such cases, the patients are enabled immediately to discontinue the use of purgative medicines; they can bear a mixed animal and vegetable diet, in the ordinary proportion; a regular action of the bowels is shortly acquired, and no further stimulant or pharmaceutical tonic is necessary. When they quit the establishment, formal and complex means being no longer required, we are assured that they are able, for a time, at least, to maintain the ground gained, simply by common-sense diet, drinking a few glasses of water in the morning, taking a daily cold-bath, and persevering in their habitual exercise. The country rings with such accounts as these; if they are correct, undoubtedly, the patients are in a fair way of recovering their lost health and strength, and are pursuing, subsequently to systematic treatment, a much more rational and scientific course of medical habits, than that enjoined to the dyspeptic disciple of medical orthodoxy.

The questions, with which we set out, may now be hypothetically answered: they were, "Does hydropathy furnish the physician with instruments which he, as a skilful workman, can undertake to employ? Does it contain, among its various machinery, any really therapeutic means, any properties capable of carrying out the indications which we regard as palpable in many diseases?" These questions, we think, may be allowed to have been answered in the affirmative, if we may depend on the results of our own limited experience; they must be allowed to be so answered, and unequivocally, if we may admit as perfectly trustworthy, the accounts published by the hydropathists themselves, and by those who have subjected themselves to the treatment. On another occasion we may, perhaps, endeavour to sift this evidence in a more rigid manner, in order to ascertain, with certainty, what in it is true, what false, what doubtful, and what inapplicable. But in any inquiry we may institute, we must continue to examine the water-cure relatively to other modes of practice: this is the only method of arriving at an estimate of its actual value to the practical physician. The imperfections which it shares in common with ordinary treatment, and which are inseparable from all human performances, may be left entirely out of sight; to dwell on them, would be uselessly to encumber the question, like inserting a crowd of corresponding items upon both sides of an equation. The philosopher's duty is to remove such superfluities, in order that the real problem may appear in a just and intelligible form.

In conclusion, we will venture to place on record the following, as among the more important impressions which have remained on our mind after a careful examination of the whole subject:

1. We should be glad to see Dr. Currie's practice revived (for the sake of experiment, at least,) in all its boldness, for the suppression of the general febrile paroxysm. On carefully looking over the evidence published by Dr. Currie and his contemporaries, it is impossible to deny that they attained a larger amount of success in treating fever by water than other practitioners have done by other means. We have already pointed out how their practice has been misunderstood by modern writers. But, while we regard this practice as well adapted for treating general fever, we find no proof that it is competent to meet the dangerous local complications with which fever is so often accompanied. These complications may reasonably be expected less frequently, when the early treatment of fever is rendered more efficacious. But, when they do occur, we find nothing in hydropathic writers to show that lancets, leeches, blisters, &c., can be dispensed with.

2. In a large proportion of cases of gout and rheumatism the water-cure seems to be extremely efficacious. After the evidence in its favour accessible to every body, we think medical men can hardly be justified in omitting—in a certain proportion of cases, at least—a full trial of it. No evidence exists of any special risk from the water-practice in such cases.

3. In that very large class of cases of complex disease, usually known under the name of chronic dyspepsia, in which other modes of treatment have failed or been only partially successful, the practice of Preissnitz is well deserving of trial.

4. In many chronic nervous affections and general debility we should anticipate great benefit from this system.

5. In chronic diarrhea, dysentery, and hemorrhoids, the sitz-bath appears to be frequently an effectual remedy.

6. We find nothing to forbid a cautious use of drugs in combination with hydropathic measures. On the contrary, we are convinced that a judicious combination of the two is the best means of obtaining the full benefit of each. The water-cure contains no substitute for the lancet, active purging, and many other means necessary for the relief of sudden and dangerous local maladies. The banishment of drugs from his practice was necessary, and perhaps natural, on the part of Preissnitz: the like proceeding on the part of qualified medical men superintending water-establishments in this country, evinces ignorance or charlatanry, or both.

7. With careful and discreet management, in the hands of a properly qualified medical practitioner, the water-cure is very rarely attended with danger.

8. Many of the principal advantages of hydropathy may be obtained in a private residence, with the assistance of ordinary moveable baths. Therefore, it can easily be brought under the direction of the regular medical practitioner.

9. In many cases, however, it is evident that what may be termed the mere *accessories* of the water-cure, are of extreme importance in bringing about a favorable result; and these accessories are frequently not available—or available in a very inferior degree—in ordinary practice. Among the more important of these accessories we may mention the following as having relation to most of the chronic cases treated in hydropathic establishments: 1, relief from mental labours of an exhausting or irritating kind, from the anxieties and responsibilities of business, from domestic irritations of various kinds, from mental inaction or ennui, &c.; 2, change of locality, air, scene, society, diet, &c.; 3, the fresh mental stimulus involved in the almost constant occupation of the patient's time, in the performance of the numerous and various dabblings, paddlings, sweatings, washings, drinkings, rubbings, &c., imposed by the water treatment; 4, the frequent and regular bodily exercise taken in the open air, or within doors; 5, the powerful mental stimulus supplied by the confidence generally reposed by the patients in the means employed, and by the consequent hope, alacrity, cheerfulness, &c.; 6, the total abandonment of vinous and other stimulants, and of drugs,—all of which have, in a large proportion of cases, been tried and found, not only useless, but probably, productive of disadvantage.

10. A certain and not inconsiderable portion of the benefits derived from hydropathic establishments are, however, attainable without them, by other means, as by travelling, &c. &c. For example, we suspect that many of the most striking results witnessed in such establishments, as in the case of Sir Edward Bulwer Lytton or Mr. Lane, would have probably been obtained, if the patients had chosen to hire themselves, and had worked as agricultural labourers, in a dry, healthy district, and had lived on agricultural fare, sufficiently nutritious in quantity and kind, for a sufficient length of time.

11. Notwithstanding the success of the founder of hydropathy, its practice by non-professional persons can neither be fully advantageous nor safe. At the same time, it is true that very little experience is necessary to enable an educated medical man to acquire sufficient insight into it for purposes of practice. Many of the best hydropathic physicians have, in the first instance, devoted very few weeks to studying the subject in Germany.

12. Many advantages would result from the subject being taken up by the medical profession. The evils and dangers of quackery would at once be removed from it. Its real merits would soon be known. The tonic portion of its measures might then be employed in conjunction with special remedies of more activity, which, no doubt, would often prove exceedingly beneficial.

13. The benefits ascribed to hydropathy, but arising indirectly from the abandonment of drugs, vinous and other stimulants, &c., may certainly be obtained without sending patients to Graeffenberg.

14. Finally, it must always be remembered that the distinction between quacks and respectable practitioners is one, not so much of remedies used, as of skill and honesty in using them. Therefore, let our orthodox brethren be especially anxious to establish and to widen, as far as possible, this distinction between themselves and all spurious pretenders. "*Artem medicam denique videmus, si à naturali philosophia destituatur, empiricorum praxi haud multum præstare. Medicina in philosophia non fundata, res infirma est.*"

